# Methyl Cation Affinity and Methyl Anion Affinity Prediction Using Uni-Mol-Based Models

**DOI:** 10.1021/acs.jpca.5c08537

**Published:** 2026-06-16

**Authors:** Yuto Iwasaki, Akinori Sato, Tomoyuki Miyao

**Affiliations:** † Graduate School of Science and Technology, 12708 Nara Institute of Science and Technology, 8916-5 Takayama-cho, Ikoma, Nara 630-0192, Japan; ‡ Data Science Center, Nara Institute of Science and Technology, 8916-5 Takayama-cho, Ikoma, Nara 630-0192, Japan

## Abstract

Predicting nucleophilicity and electrophilicity at atomic sites in organic compounds is crucial for the design of polar reactions. Methyl cation affinity (MCA) and methyl anion affinity (MAA), calculated using quantum-chemistry-based simulations, are good indicators of nucleophilicity and electrophilicity, respectively. Machine learning surrogate models have been developed to accelerate inference for MCA and MAA. However, their prediction accuracy, even for state-of-the-art models, remains inadequate for practical use. We present accurate machine-learning surrogate models for MCA and MAA, achieving root-mean-square errors of 8.90 [kJ/mol] for MCA and 10.02 [kJ/mol] for MAA. The model architecture comprises a pretrained Uni-Mol encoder block and a feed-forward neural network. Without pretraining on massive conformers, models with the proposed architecture still perform comparably, suggesting its architectural superiority to a molecular graph-based neural network model. The conformation used to calculate MCA and MAA as the model input is found to slightly improve prediction accuracy. Furthermore, the proposed models are robust to underlying MCA/MAA calculation protocols. The inference time of the proposed MCA and MAA surrogate models is less than 0.1 s per compound on a single GPU, and data scarcity arising from expensive MCA/MAA calculation protocol can be overcome by a simple fine-tuning approach, enabling fast and accurate reactivity prediction for synthetic chemistry.

## Graphical abstract

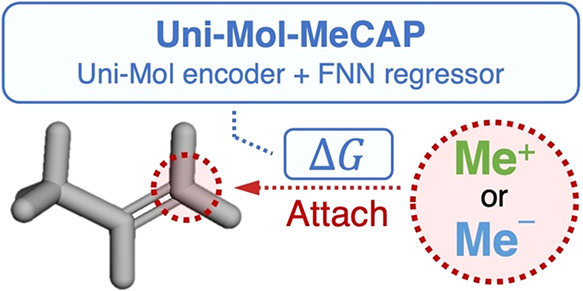

## Introduction

Predicting substrate reactivity is essential for designing organic reactions. Mayr’s nucleophilicity (*N*) and electrophilicity (*E*) parameters^1^ quantify a substrate’s tendency to act as a nucleophile and an electrophile. Deriving these parameters requires experiments with carefully controlled reaction conditions using a series of reference reactants. Therefore, in-silico methods for predicting these parameters have been extensively investigated.^2–4^ Mayr et al. originally reported that the methyl anion affinity (MAA)–calculated energy difference of methyl anion addition reaction to an electrophile–was highly correlated with the *E* parameter^5^ for carbocations. Since then, the correlation between a quantum-chemically calculated MAA and the *E* parameter has been investigated for specific chemical series under specific calculation conditions,^7^ e.g., ketones and Michael acceptors,^6^ including solvent effect in DFT calculations.^11^ On the other hand, methyl cation affinity (MCA)–calculated energy difference of methyl cation addition reaction to a nucleophile^9,10^ —was recently found to be correlated with the *N* parameter (*Ns_N_*).^8^ Thus, MCA and MAA can be considered reliable indicators of kinetic nucleophilicity and electrophilicity, respectively.

MCA and MAA for neutral nucleophile (Nu) and electrophile (El) are defined as

1 $$\mathrm{MCA}=E\left(\mathrm{Nu}+{\mathrm{CH}}_{3}^{+}\right)-E\left({\mathrm{Nu}}^{+}{\text{−}\mathrm{CH}}_{3}\right)$$

2 $$\mathrm{MAA}=E\left(\mathrm{El}+{\mathrm{CH}}_{3}^{-}\right)-E\left({\mathrm{El}}^{-}{\text{−}\mathrm{CH}}_{3}\right)$$

where *E*(X) represents the energy for component *X*. The standard Gibbs free energy or the standard formation enthalpy is commonly used as an energy measurement. Mayr’s original formulation for MCA and MAA assumes a gas phase, and these parameters are correlated with the N and E parameters if the substrates consist of the same functional group. Computing these quantities in the solvation model (MCA*, MAA*) reduced the functional-group dependence and yielded a single, broadly applicable linear correlation with Mayr’s parameters under aprotic conditions.^8,11^ Hereafter, we refer to the MCA* and MAA* as MCA and MAA, respectively, for simplicity. As a reliable, broadly applicable, and fast computational method, Ree et al. proposed an automated MCA and MAA calculation workflow, starting with nucleophilic and electrophilic site identification using substructure matching of rule-based reactive sites with the RDKit library,^12^ followed by conformational search and structure optimization with GFN1-xTB ALPB (DMSO), and evaluating energies with a r^2^SCAN-3c single point DFT calculation under the SMD (DMSO) solvation model.^13^ The calculated MCA and MAA were found to be highly correlated with Mayr’s scales (*R* ^2^ = 0.84 for *Ns*_*N*_ and 0.94 for *E*), with a median wall time of less than 2 min per molecule on commodity CPUs.

To further speed up MCA and MAA calculation for evaluating millions of substrates during in-silico screening, machine learning (ML)-based surrogate models were proposed.^14,15^ These models predict MCA and MAA from a molecular structure without reaction simulation. Ree et al. trained a LightGBM ML model using approximately 50,000 neutral drug-like molecules, with MCA and MAA annotations calculated through the automated pipeline they developed.^15^ The model uses atom-based features of the charge model 5 (CM5) atomic charges surrounding a target atomic site, resulting in prediction accuracy values of *R* ^2^: 0.94, RMSE: 17.45 [kJ/mol] for MCA, *R* ^2^: 0.91, RMSE: 22.08 [kJ/mol] for MAA.

Although these surrogate models (terms LightGBM with CM5 charge models) reported relatively high prediction accuracy for MCA and MAA, their accuracy is inadequate for practical application. In chemo-selectivity identification or subproduct prediction, two or more MCA or MAA values in reactants are compared, which doubles the uncertainty of the prediction model. For example, roughly estimating and assuming no correlation in errors between samples, an RMSE of 22.08 [kJ/mol] gives an uncertainty of 44.16 [kJ/mol] for a pair of MAA values,^15^ meaning that ranking reactivity sites based on the model is reliable only when the difference for them exceeds this error.

We previously reported that Uni-Mol^16^—an SE(3)-equivariant transformer architecture—models predicted conformation-dependent properties with high accuracy, whereas using inaccurate conformers resulted in lower prediction accuracy.^17^ This suggests that Uni-Mol models may predict properties in a chemically meaningful way, rather than simply fitting to a training data set. Furthermore, it also achieved high prediction accuracy on several chemically relevant tasks, including ^13^C chemical-shift^18^ and p*K*_a_ prediction.^19^ Regarding molecular features used in the previously proposed surrogate ML models, CM5-based features were limited to local information around an atomic site for MCA or MAA prediction.

Here, we develop Uni-Mol-based MCA and MAA surrogate models, termed Uni-Mol-MeCAPs (Methyl Cation/Anion affinity Predictors), to achieve high prediction accuracy for reaction-site comparison applications. The Uni-Mol-MeCAPs are atom-based fine-tuning models consisting of a pretrained Uni-Mol model and a simple feed-forward network (FFN) that takes an entire molecular conformer as input. These models are compared with the models proposed by Ree et al.,^15^ and ChemProp models,^20^ which are topological molecular graph-based neural networks. Through a rigorous validation of predictability and the importance of conformation for a Uni-Mol-MeCAP model input, we aim to demonstrate that Uni-Mol-MeCAP-Cat (Cation) and Uni-Mol-MeCAP-An (Anion) are reliable for MCA and MAA prediction. The outputs of the Uni-Mol-MeCAPs are interpreted by examining the attention weights, and the usefulness of the models are demonstrated using well-studied chemical reactions. As a practical approach to calibrating the Uni-Mol-MeCAPs against higher-level yet computationally demanding QM-based energies for optimized conformations, a simple fine-tuning method is proposed and validated using a test data set.

Scripts to construct Uni-Mol-MeCAPs and to reproduce the results in this study are available in a public repository.

## Materials and Methods

### Uni-Mol-MeCAP

#### Overview

A schematic for MCA prediction at a specified atomic site using the proposed Uni-Mol-MeCAP-Cat is summarized in Figure 1. The same workflow is used for the MAA prediction model: Uni-Mol-MeCAP-An. Given a target atomic site (e.g., the phenol oxygen of paracetamol in Figure 1a) of a molecule, a conformer is generated using the ETKDGv3 method implemented in the RDKit library, followed by the optimization under the MMFF94 force field (Figure 1b); if conformer generation fails, the coordinates of a two-dimensional flattened structural formula are used instead. Two versions of the Uni-Mol encoder blocks are tested: Uni-Mol1^16^ and Uni-Mol2.^21^ Their differences will be explained in the subsequent section. The model’s input consists of a set of elements and their Cartesian coordinates. When the Uni-Mol2 modeling architecture is employed as an encoder, hydrogen atoms are suppressed, and atom and bond attributes (such as chirality, hybridization, formal charge, and bond order) are also provided as input (Figure 1c). The output of the Uni-Mol’s encoder block (Figure 1d) consists of atom-wise vectors (atom representation), and the vector for the target atom is input to the FFN (Figure 1e and f), obtaining the predicted MCA for the atom.

**Figure 1:**
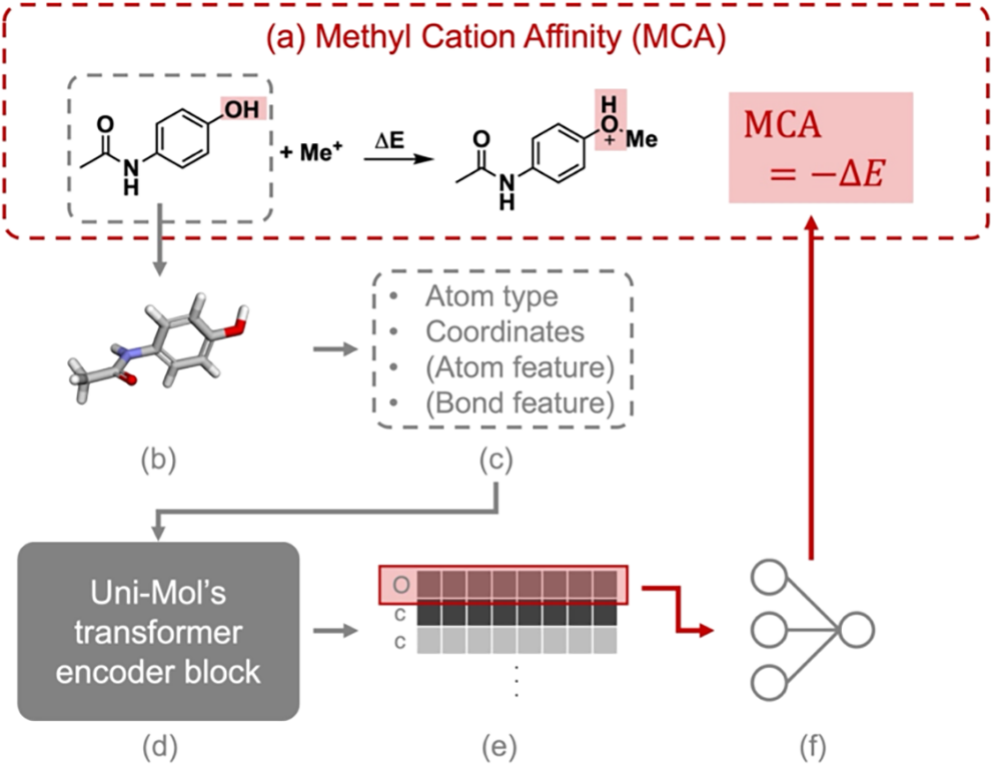
Methyl cation affinity (MCA) prediction using the Uni-Mol-MeCAP-Cat. (a) MCA is defined as the negative value of the change in the energy of the methyl cation addition reaction: −Δ*E*. (b) Conformers are generated and optimized with the RDKit library using the ETKDGv3 and the MMFF94 force field; if conformer generation fails, the coordinates of a two-dimensional flattened molecular graph are used instead. (c) The model’s input comprises elements and atomic coordinates; for the Uni-Mol2 encoder block, atom and bond features are also included. (d) The inputs are fed into a pretrained Uni-Mol transformer encoder block to obtain atom-wise embeddings. (e) The embedding corresponding to the target atom is selected, and (f) the target-atom embedding is passed to a feed-forward network (FFN) to predict its MCA. The same procedure is used to predict the methyl anion affinity (MAA).

#### Uni-Mol’s Transformer Encoder Block

There are two versions of Uni-Mol: version 1 (Uni-Mol1)^16^ and version 2 (Uni-Mol2).^21^ The encoder block in either version is used in a Uni-Mol-MeCAP. Although the encoder architectures differ in several respects between Uni-Mol1 and Uni-Mol2, both share the basic design principle of using atom and atom-pair representations and updating them via attention layers. The atom-pair representation encodes the geometric (distance) relation between atoms via a kernel function. Uni-Mol2 further incorporates topological and bond information into the atom-pair representation and explicitly updates pair representations using OuterProduct and TriangularUpdate operations.^21,22^ Unlike LightGBM with CM5-charge models, the Uni-Mol encoder operates directly on learned molecular representations and does not explicitly use atomic charges. The model’s complexity, measured by the number of tunable parameters, varies: the Uni-Mol1 model used in this study has approximately 48 million parameters, while the Uni-Mol2 model has about 84 million. For both models, the atom-wise embeddings from the encoder output are used for downstream MCA or MAA prediction (Figure 1e). Both Uni-Mol encoder blocks were pretrained prior to integration into Uni-Mol-MeCAPs, using atom-masking prediction and 3D coordinate recovery (denoising pretraining) techniques.^23^ For pretraining, large-scale molecular data sets were prepared. For Uni-Mol1, an unlabeled molecular corpus assembled from two sources was prepared: approximately 12 million purchasable compounds curated from a commercial vendor collection and molecules gathered in a prior work using the ZINC^24^ and ChEMBL^25^ databases. Normalization and deduplication across the molecules in the combined sources yielded approximately 19 million unique molecules. For each molecule, 11 conformers, including a two-dimensional flat molecular graph, were generated with the ETKDGv3 generator and optimized under the MMFF94 force field using the RDKit library, yielding approximately 209 million conformers. For Uni-Mol2, a larger pretraining data set was prepared. The authors integrated the Uni-Mol1 pretraining data set with the ZINC20 standard data set as downloaded using the “standard reactivity tranche” query, consisting of approximately 884 million compounds. The number of ETKDGv3-based conformers for pretraining was approximately 838 million obtained by the temperature-based sampling method, and they were optimized using the RDKit library under the MMFF94.

To assess the effect of using pretrained Uni-Mol encoders on prediction accuracy, we train Uni-Mol-MeCAPs from scratch, without loading the pretrained Uni-Mol weights. All other settings, including the model architecture, data split, hyperparameters, and training protocol, are kept identical to those used for the pretrained Uni-Mol-MeCAPs.

#### FFN for MCA and MAA Prediction

The FFN for MCA and MAA takes a 512-dimensional (For Uni-Mol1) or 768-dimensional (For Uni-Mol2) input vector for a target atomic site from the Uni-Mol encoder block and outputs a scalar value. The number of hidden layers was optimized by testing values from 0 to 2. Each hidden layer comprises 512 input and output units, with GELU activation, and a dropout rate of 0.1.

#### Fine-Tuning

All parameters in the Uni-Mol encoder block and the FFN layer of the Uni-Mol-MeCAP are optimized during training (fine-tuning). The loss function is the mean-squared error (MSE) on the scaled MCA or MAA, computed using the training data set. Let *y*_*i*_ denote the QM-derived MCA or MAA value for an atomic site *i*, *ŷ*_*i*_ be the model prediction, and *N* the total number of training atomic sites for MCA or MAA. *y*_*i*_ is standardized using the mean value μ_train_ and standard deviation σ_train_ of *y* for the training data set as

3 $$y_{i}^{\prime }=\frac{y_{i}-\mu_{\mathrm{train}}}{\sigma_{\mathrm{train}}}$$

The loss is computed on the standardized scale

4 $$L_{\mathrm{MSE}}=\frac{1}{N}\sum_{i=1}^{N}{\left({{ŷ}_{i}}^{\prime }-{y_{i}}^{\prime }\right)}^{2}$$

Since the training is based on atomic sites for MCA and MAA, there may be a bias toward compounds with many MCA or MAA sites. The models are trained for 50 epochs using an AdamW optimizer (learning rate of 1 × 10^–4^, weight decay of 0.01, batch size of 50).^26^ Evaluation metrics are reported after scaling back to the original physical scale using the inverse transformation.

#### Interpretation of the Uni-Mol-MeCAPs

Prediction outcomes of the Uni-Mol-MeCAPs can be interpreted on structural formula and atomic coordinates by inspecting the self-attention weights of the Uni-Mol encoder block since atomic interaction does not occur in the FFN block of the predictors.

In the self-attention mechanism of the Uni-Mol encoder block, a pair representation is incorporated as a bias term in addition to the standard scaled dot-product attention. The attention weight matrix **S** ^l,h^ at the *l*-th layer and *h*-th head is defined as follows:

5 $$S^{l,h}=\mathrm{softmax}\left(\frac{Q^{l,h}{\left(K^{l,h}\right)}^{T}}{\sqrt{d}}+D^{l-1,h}\right)$$

where **Q** ^l,h^ and **K** ^l,h^ denote the query, key, and value matrices at the *l*-th layer *h*-th head, respectively; *d* is the dimensionality of the hidden representations; and **D** ^l–1,h^ is the pair representation obtained from the preceding layer, which serves as a bias term encoding three-dimensional spatial information between atom pairs. To obtain a single aggregated attention weight matrix, we averaged the attention weights across all heads

$$S^{l}=\frac{1}{H}\sum_{h=1}^{H}S^{l,h}$$ 6

where *H* denotes the total number of attention heads. In this study, only the attention weights from the final layer were used.

In the Uni-Mol encoder block, CLS and EOS tokens are also included in the computation of attention weights; however, since these tokens do not correspond to actual atoms, the rows and columns corresponding to them were removed from **S** ^l^ before calculating atomic contribution. Subsequently, to evaluate the importance of atom *j* within the molecule, the *j*-th row (**S** *j* ^l^) was extracted from **S** ^l^. The *j*-th row represents the attention distribution of all atoms for atom *j*. It should be noted that because the attention weights for CLS and EOS have been removed from **S** ^l^, the sum of the atomic contributions does not equal one. The resulting per-atom attention scores were mapped onto the corresponding molecular structure and visualized using a continuous color scale.

### MCA and MAA Prediction Models for Comparison

#### LightGBM with CM5 Charge Model

LightGBM with CM5 charge is the state-of-the-art for MCA and MAA prediction proposed by Ree et al.^15^ Their models predicted MCA and MAA using handcrafted and per-site descriptors built from the CM5 partial charges computed with xTB.^27–29^ Each record corresponds to one molecule and atomic site, and the models learn direct mappings from these features to MCA and MAA values. In this study, we reused the trained models provided by the authors, which we confirmed can fully reproduce the reported statistics.^15^

The comparison between our models and the LightGBM with CM5 charge models is strictly valid for the reference split defined in their publication, except for the conformers, which we believe are negligible (see the *MCA and MAA data set* section).

#### ChemProp Model

To compare a two-dimensional molecular representation with the 3D-based neural network approach, a ChemProp implementation of the directed message-passing neural network model^20^ was used. After *L* rounds of directed-bond message passing on standard ChemProp atom/bond features, we obtain per-atom embedding *h*_i_ for the *i*-th atom, and the embedding of the designated target atom *h*_target_ (indexed through template-based substructure matching in preprocessing) is fed into a linear head (no hidden layers) to predict MCA or MAA. Similar to fine-tuning for Uni-Mol-MeCAP, all parameters are optimized. The loss function is the MSE on standardized MCA or MAA values. Models are trained for 50 epochs using the AdamW optimizer (learning rate of 1 × 10^–4^, weight decay of 0.01, batch size of 50). Evaluation metrics are reported on the original physical scale after inverse transformation of predictions for reporting purposes only. The MPNN layer was not pretrained, unlike the Uni-Mol encoder blocks.

### Data Sets and Evaluation Strategy

#### MCA and MAA Data Set

The data set of approximately 50,000 drug-like small molecules, annotated with MCA and MAA, reported by Ree et al.,^15^ was used. We downloaded and extracted parts of the data set from the reference^15^ and organized it into tuples of SMILES, atom index, and QM-derived MCA or MAA, where the atom index indicates the position of the methyl cation or methyl anion attachment. QM-derived MCA or MAA values were calculated from the xTB-optimized conformations of the input compound and output product (methyl cation (anion) attached product). These annotated values were reused in this study. As a data splitting strategy, two protocols were tested:

1. **The reference split**: the original partitioning reported by Ree et al. We used fold 1 as the validation set, with the remaining folds used for training. The test set is identical to that used in the reference.^15^
2. **The scaffold-based split**: distinct compound-based partitioning based on molecular scaffolds. Bemis–Murcko frameworks^30^ are used as scaffolds. Compounds with the same scaffold were assigned to one of the training, validation, and test sets. Thus, any compound with multiple methyl cation or methyl anion attachment sites existed only in one of the sets, no leakage in chemical structures was assured. For molecules without a scaffold (e.g., acyclic structures), scaffolds were defined as themselves. These scaffolds were grouped by canonical SMILES and split as such that the corresponding number of compounds satisfied the following ratios: 15% of all compounds for the external test set, and the remaining 85% were for training (80% of the remaining) and validation (20%).

The number of entries (atomic sites for MCA or MAA) for the two data-splitting strategies is summarized in Table 1. Model prediction accuracies are evaluated in terms of the coefficient of determination *R* ^2^, the Pearson coefficient *R* between QM-derived and predicted MCA or MAA values, and the root-mean-square error (RMSE).

**Table 1:** Number of MCA and MAA Entries for Two Split Strategies^a^

		Reference split		Scaffold-Based split	
LightGBM model with CM5 charge	Uni-Mol and ChemProp models	MCA	MAA	MCA	MAA
Training	Validation	110,646	90,801	111,951	92,405
	Training	442,582	363,200	440,645	361,605
Test	Test	97,629	80,118	98,261	80,109

a The number of entries (MCA or MAA values) for each fold is reported. The reference split is the one used by Ree et. al.^15^ (we determined the number of entries from the downloaded dataset), while the scaffold-based split ensures non-overlap in molecular scaffolds between training and test datasets. For the LightGBM model with CM5 charge, no validation dataset was used.

#### Conformation Generation

Regarding conformation, we used RDKit-based conformers (generated by ETKDGv3 and then optimized by MMFF94) as input to the tested models, unless otherwise specified, due to their low computational cost. We further assessed the relationship between conformation quality and the models’ prediction accuracy, including xTB-optimized conformations.

#### QM Protocol Comparison

Because MCA and MAA are protocol-dependent QM-derived descriptors, we assessed the sensitivity of MCA/MAA regression to the electronic-structure reference scheme. For this purpose, additional data sets with MCA and MAA derived across multiple QM protocols were prepared as follows.

Starting from 10,000 neutral molecules (total formal charge = 0) randomly drawn from the original MCA/MAA data set,^15^ we uniformly sampled one MCA site and one MAA site from the corresponding candidate sets and recorded the selected atom indices. These atom indices were used for all QM protocols described below. For each molecule and its fixed MCA and MAA atom indices, we constructed the corresponding methylated adducts and computed affinities using identical target definitions across protocols.

We compared the following reference schemes (software and versions in parentheses), using the standard “A//B” notation to denote single-point energies at level A evaluated on geometries optimized at level B:

1. **Proposed protocol (our main reference):** r^2^SCAN-3c//GFN1-xTB. Geometries were optimized at GFN1-xTB with ALPB­(DMSO) using xTB (version 6.5.1), and single-point energies were computed at r^2^SCAN-3c with SMD­(DMSO) using ORCA (version 5.0.1).^15^
2. **Higher-level DFT protocol**: wB97-xd/def2-TZVP//r^2^SCAN-3c. Geometries were reoptimized at r^2^SCAN-3c with SMD­(DMSO) starting from the structures obtained in (1), and single-point energies were computed at ωB97X-D/def2-TZVP with SMD (DMSO). ORCA (version 6.1.1)^31,32^ was used for geometry optimization and Gaussian 16 ^33^ for single-point calculation.
3. **Lower-level semiempirical protocol**: PM7//PM7. Geometries were reoptimized at PM7 starting from the structures obtained in (1), and their energies were calculated at PM7 with EPS = 46.826 using MOPAC (version 23.2.3).^34^

Due to computational resource constraints, we resampled 5000 molecules from those for which the ORCA-based geometry optimization in (2) succeeded, and the molecular connectivity was verified as correct (*rdDetermineConnectivity* provided by RDKit was used for this verification). To ensure a controlled protocol-to-protocol comparison, we further retained only molecules for which the selected MCA/MAA sites were successfully computed under all compared protocols and for which the descriptor set required by the LightGBM baseline of Ree et al.^15^ is stored. The final data set for protocol-to-protocol comparison comprises 4,827 compounds (train/validation/test = 3,827/500/500).

### Software and Model Implementation

Scripts for all experiments were written in Python using PyTorch 2.7.1,^35^ PyTorch Geometric 2.6.1,^36^ and torch-scatter 2.1.2.^37^ We utilized the *unimol_tools* library at version 0.1.4.post1 ^38^ to load the pretrained Uni-Mol1 or Uni-Mol2 checkpoints and for preprocessing or extracting representations. Cheminformatics preprocessing—SMILES parsing, MMFF94-based conformer optimization, canonicalization, and SMARTS-based site annotation—was conducted using RDKit 2025.9.1.^12^ Experiments were conducted on a Linux computer with Ubuntu 22.04, one Intel­(R) Xeon­(R) Platinum 8480+ CPU and eight NVIDIA H100 80GB HBM3 GPUs, using CUDA 12.9 as the GPU library. For comparison models, experiments were designed according to the instructions provided in the official repository. Scripts to construct Uni-Mol-MeCAPs and to reproduce the results in this study are available in the online repository at https://github.com/iwmspy/MeCAP

## Results and Discussion

### Accuracy of Uni-Mol-MeCAPs

#### The Reference Split

Table 2 presents the prediction accuracy for the test data set of the reference split. The number of FFN layers in the Uni-Mol and ChemProp models was set to zero, as discussed in “Hidden Layer Optimization for the FFN in the Uni-Mol-MeCAPs”. The best epoch was identified as the one with the lowest validation loss. In Table 2, the best epoch, the coefficient of determination: *R* ^2^, the Pearson correlation coefficient R between QM-derived and predicted *y* values, and RMSE [kJ/mol] are reported. On this data set, Uni-Mol-MeCAPs significantly outperformed LightGBM using the CM5 charge and ChemProp models. For MCA, the Uni-Mol2-based MCA prediction model (Uni-Mol2-MeCAP-Cat) achieved an RMSE of 8.90 [kJ/mol], while the state-of-the-art LightGBM with CM5 charge model reached an RMSE of 17.45 [kJ/mol]. Likewise, for MAA, the Uni-Mol2-based MAA prediction model (Uni-Mol2-MeCAP-An) achieved an RMSE of 10.02 [kJ/mol], while the LightGBM with CM5 charge model reached an RMSE of 22.08 [kJ/mol].

**Table 2:** Accuracy of MCA and MAA Prediction Models for the Test Dataset of the Reference Split^a^

Target	Model	Best epoch	*R* ^2^	Pearson R	RMSE [kJ/mol]
MCA	Uni-Mol1-MeCAP-Cat	48	0.98	**0.99**	9.87
	Uni-Mol1-MeCAP-Cat (without pretraining)	45	0.98	**0.99**	9.71
	Uni-Mol2-MeCAP-Cat	50	**0.99**	**0.99**	**8.90**
	Uni-Mol2-MeCAP-Cat (without pretraining)	46	0.98	**0.99**	9.43
	ChemProp	50	0.91	0.95	21.64
	LightGBM with CM5 charge^15^	-	0.94	0.97	17.45
MAA	Uni-Mol1-MeCAP-An	48	**0.98**	**0.99**	10.99
	Uni-Mol1-MeCAP-An (without pretraining)	43	**0.98**	**0.99**	10.98
	Uni-Mol2-MeCAP-An	50	**0.98**	**0.99**	**10.02**
	Uni-Mol2-MeCAP-An (without pretraining)	44	**0.98**	**0.99**	10.63
	ChemProp	50	0.83	0.91	29.68
	LightGBM with CM5 charge^15^	-	0.91	0.95	22.08

a The number of epochs for the Uni-Mol and ChemProp models was determined based on the validation loss. For each model and target, the best epoch, the *R* ^2^, Pearson *R*, and RMSE are reported. The results of LightGBM with CM5 charge were replicated from ref 15 under CC BY 3.0 license. The highest accuracy for each target is highlighted in bold.

Surprisingly, without pretraining (Table 1 for Uni-Mol-MeCAPs (without pretraining)), Uni-Mol-MeCAPs performed comparably to the pretrained counterparts. For example, the pretrained Uni-Mol2-MeCAP-Cat achieved an RMSE of 8.90 [kJ/mol] while without pretraining this architecture achieved an RMSE of 9.43 [kJ/mol]. This underscores the importance of Uni-Mol’s architecture: a 3D transformer that incorporates geometric information and suggests that pretraining on massive conformers was unnecessary. In this study, the number of training samples was relatively large: 442,582 and 363,200 for the MCA and MAA reference splits, respectively. The weights of Uni-Mol-MeCAPs might be effectively tuned without starting from pretrained weights. Another possible reason is that the MCA and MAA predictions may not be associated with the pretraining tasks used by the Uni-Mol models, since MCA and MAA represent the energy differences before and after methyl group attachments to a molecule.

Since prediction accuracy, as measured by RMSE, was similar between the Uni-Mol1- and Uni-Mol2-based models (Table 2), we conducted paired *t* tests on the test-set prediction errors. The details of the paired analysis are provided in Section S1 of the Supporting Information. This analysis revealed a consistent advantage of Uni-Mol2-MeCAPs over Uni-Mol1-MeCAPs. The mean error differences were 0.80 [kJ/mol] for MCA and 0.80 [kJ/mol] for MAA; the corresponding 95% confidence intervals were 0.76–0.83 and 0.76–0.84 [kJ/mol], respectively.

The predicted vs observed plots in Figure 2 illustrate differences in all prediction outcomes for the test data set between Uni-Mol-based and the LightGBM with CM5 charge models. In Figure 2a, orange dots represent Uni-Mol-based results, while light greendots represent LightGBM results. For both MCA and MAA, orange dots predicted by Uni-Mol-based models more closely align with the diagonal lines. Uni-Mol2-MeCAPs (**bottom row in** Figure 2a) seem slightly better than Uni-Mol1-MeCAPs (**top row in** Figure 2a) in the alignment, consistent with the statistics in Table 2. Two outliers by the Uni-Mol1-MeCAP-Cat are numbered in Figure 2a, and the corresponding structural formulas are shown in Figure 2b. The target atomic sites were the carbon atom of thiourea (compound 1 in Figure 2) and the central atom of allene (compound 2 in Figure 2). In the training data set, the central carbon of the thiourea substructure was present at 8 out of 442,582 atomic sites, and the central carbon of the allene substructure was present at 65 atomic sites. This extremely low frequency might explain the outliers produced by the Uni-Mol-MeCAP-Cat models, as they used atom types (elements) as input.

**Figure 2:**
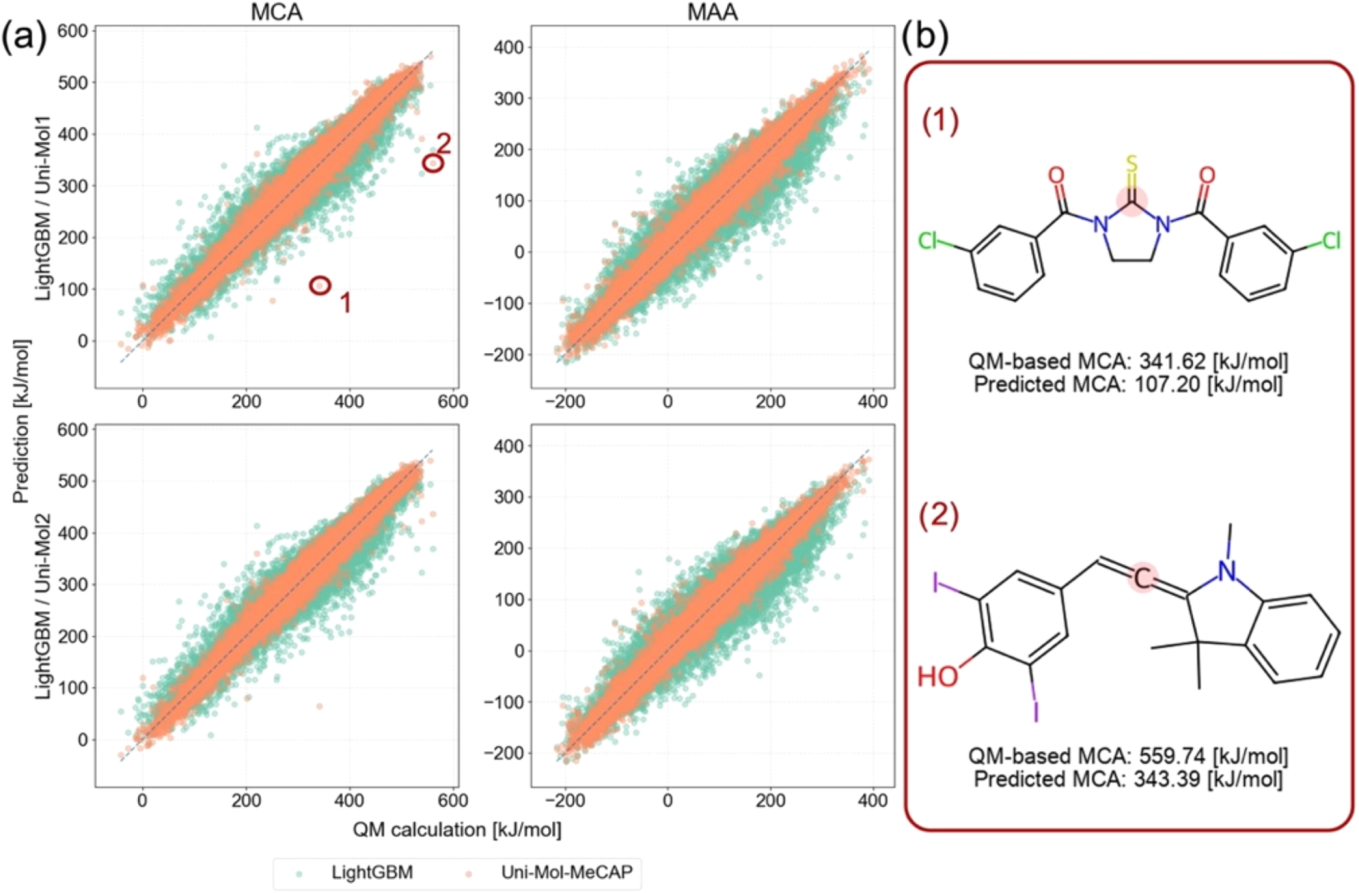
Comparison of the prediction results between Uni-Mol-based models and LightGBM models with CM5 charge. (a) The predicted vs observed plots (*y*–*y* plots) for MCA (left column) and MAA (right column) using Uni-Mol1-based models (top row) and Uni-Mol2-based models (bottom row) are shown. Each dot represents an atomic site for MCA or MAA. The Uni-Mol prediction results are marked in orange, while the LightGBM prediction results are marked in light green. The two outlier atomic sites predicted by the Uni-Mol1-MeCAP-Cat are numbered 1 and 2, and the corresponding structural formulas and atomic sites (red-filled circles) are provided in (b).

Inference speed of a Uni-Mol1-MeCAP was approximately 0.10 [sec/compound] on a computer with CPU: Intel­(R) Xeon­(R) CPU E5–2683 v4 @ 2.10 GHz (x2), GPU: NVIDIA Tesla V100-SXM2–16GB, RAM: 384GB. Thus, the inference speed of the Uni-Mol-MeCAPs is comparable with that of the LightGBM models when a GPU is employed.

#### Interpretation of Uni-Mol1-MeCAPs

We highlighted the attention weights for atomic sites that were poorly predicted by the LightGBM models with CM5-charge descriptors but were accurately predicted by the Uni-Mol1-MeCAPs, and the results are presented in Figure 3. These target atomic sites were located in conjugated systems, and a common pattern observed among the four structural formulas was that relatively high attention was assigned to atoms participating in the same conjugated system. This implies that the Uni-Mol1-MeCAPs captured chemically meaningful nonlocal structural and electronic context around the target atom, particularly delocalized electronic effects associated with conjugated systems.

**Figure 3:**
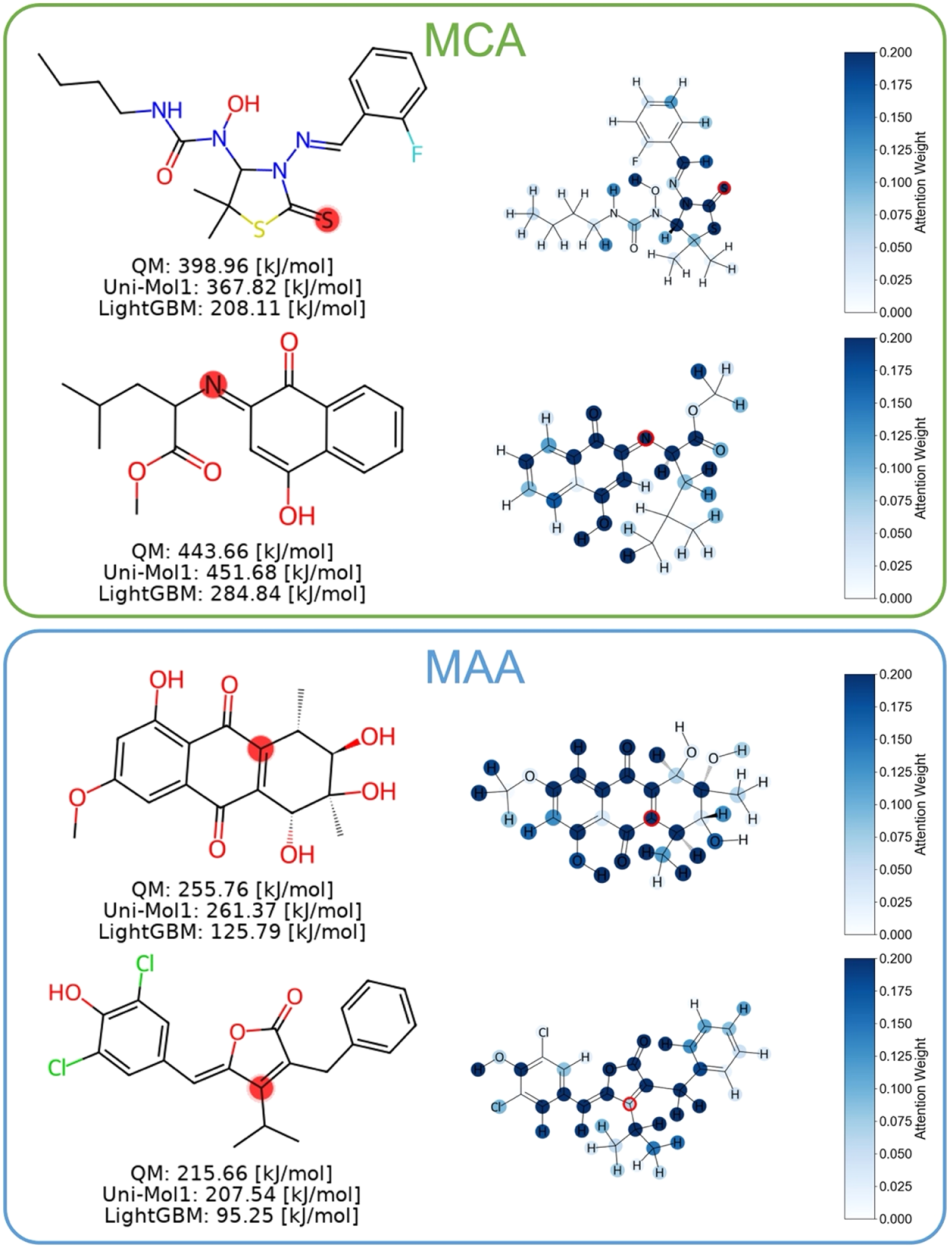
Visualization of attention weights of Uni-Mol1-MeCAPs for unsuccessful MCA/MAA predictions by LightGBM with CM5 charge model. For MCA (top) and MAA (bottom), prediction results for two example prediction targets, where the atomic sites are highlighted in red, are shown on the left, and the corresponding attention weights are color-scaled on the structural formula on the right.

#### Scaffold-Based Split

Table 3 shows prediction accuracy of Uni-Mol1-MeCAPs for the test data set of the scaffold-based split (Table 1). The scaffold-based split ensured that the training and test data sets did not contain overlapping scaffolds (and compounds also). As expected, the Uni-Mol-MeCAPs showed lower accuracy than for the reference split.

**Table 3:** Accuracy Comparison of Uni-Mol1-MeCAPs under Different Data Set Split Strategies^a^

Target	Split strategy	Best epoch	*R* ^2^	Pearson R	RMSE [kJ/mol]
MCA	Reference	48	**0.98**	**0.99**	**9.87**
	Scaffold	49	**0.98**	**0.99**	11.52
MAA	Reference	48	**0.98**	**0.99**	**10.99**
	Scaffold	44	0.97	0.98	13.23

a Uni-Mol1-MeCAPs were used for comparison. The number of hidden layers in the regression head of Uni-Mol-MeCAPs was set to 0. The highest prediction accuracy values are in bold..

To quantify and interpret this degradation for genuinely unseen scaffolds, we analyzed the test predictions along two axes: (i) scaffold-level similarity to the training set and (ii) the distance of the local atomic environment of each prediction site from the training distribution. For the scaffold-level axis, we computed 2048-bit Morgan fingerprints by RDKit^12^ for each scaffold and defined scaffold similarity as the maximum Tanimoto similarity to any scaffold in the training set. For the local-environment axis, we featurized each atomic site with a 40-dimensional “atomic environment fingerprint” and measured its Mahalanobis distance to the training distribution. The fingerprint encoded (a) center-atom descriptors (atomic number, aromaticity, ring membership, total degree, total hydrogen count, and formal charge, 6-dimensions) and (b) compositional and bonding summaries over the 1-hop and 2-hop neighborhoods, including element counts over a fixed element set [C, N, O, S, P, F, Cl, Br, I, B, Si], counts of aromatic and ring atoms, and counts of bond-type [single, double, triple, aromatic] across the corresponding shells (17-dimensions for each).

We then constructed a two-dimensional error landscape by binning test sites into quantile-based bins along these two axes (*x*: maximum scaffold Tanimoto similarity; *y*: atomic-environment Mahalanobis distance) and visualizing the median absolute error in each bin (Figure 4). This analysis revealed that when the local atomic environment was close to the training distribution (i.e., small *y* values), the prediction errors were largely insensitive to scaffold similarity. In contrast, when the local atomic environments were far from the training distribution (i.e., large *y* values), lower scaffold similarity (i.e., small *x* values) was associated with larger MAE. Taken together, these results suggest that poor performance on “unseen scaffolds” was not driven by scaffold novelty alone. Rather, prediction becomes substantially more challenging when scaffold novelty coincides with genuinely out-of-distribution local atomic environments—i.e., when both scaffold similarity is low and the site-level environment is atypical relative to the training data.

**Figure 4:**
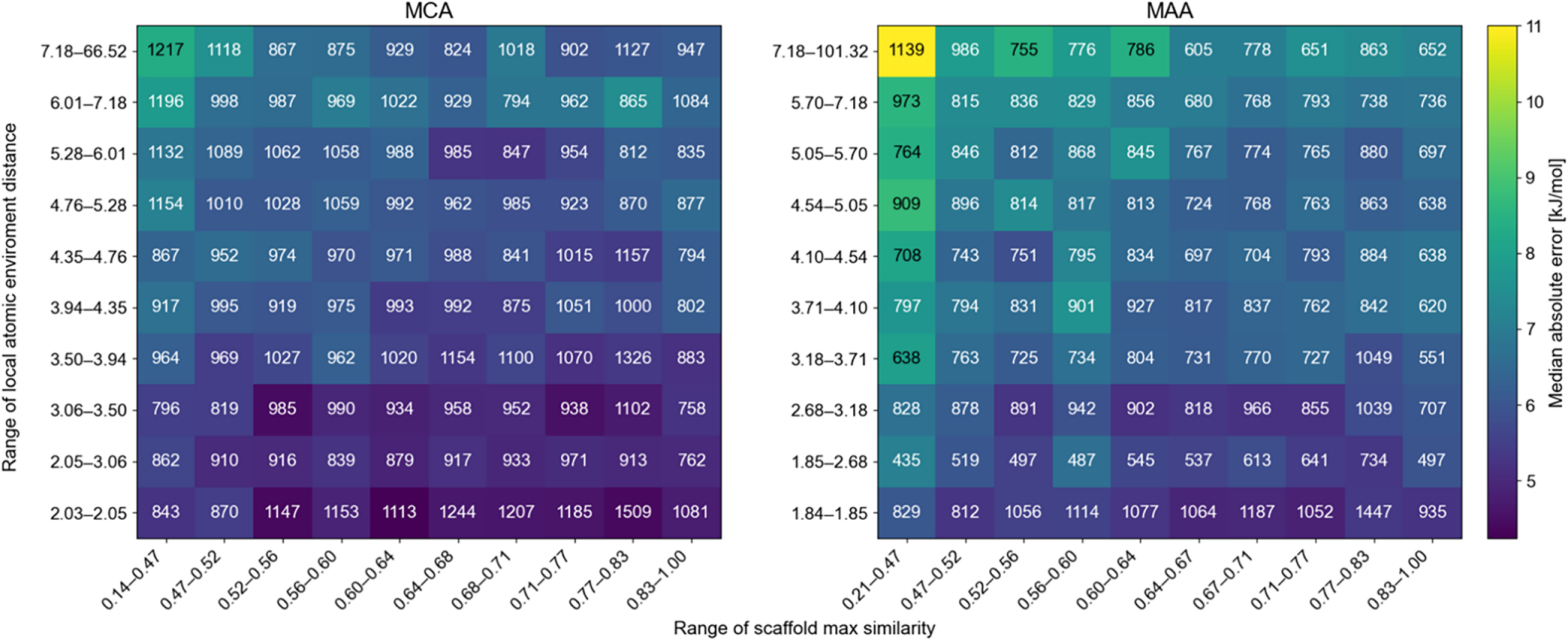
Two-dimensional error landscapes for site-level MCA and MAA predictions under the scaffold-based split. Test atomic sites were binned into quantile-based bins along two axes: (*x*) maximum scaffold-level similarity to the training set, defined as the maximum Tanimoto similarity between 2048-bit Morgan fingerprints (RDKit), and (*y*) local atomic-environment distance to the training distribution, defined as the Mahalanobis distance of a 40-dimensional atomic environment fingerprint. Each heatmap cell reports the median absolute error averaged over sites in the corresponding bin. The number in each cell represents the sample counts.

To derive simpler criteria for estimating prediction error for unseen substrates, we further examined the relationship between prediction error and the training-set frequency of the local atomic environment identical to that of a target atomic site. Each target atom with its environment was represented by a radius-1 Morgan fingerprint hash (obtained by *rdkit.Chem.rdFingerprintGenerator.GetMorganGenerator*), and target atoms in the test set were grouped based on the occurrence frequency of their corresponding hashes in the training set. For both MCA and MAA, atoms with hashes absent from the training set showed the largest median absolute errors (MCA ≃ 18.0 [kJ/mol]; MAA ≃ 18.0 [kJ/mol]). The error decreased sharply once the corresponding local environment was found in the training set and remained substantially lower for atoms with frequently observed environments (MCA ≃ 5 [kJ/mol]; MAA ≃ 5.5 [kJ/mol]), as shown in Figure S1. In other words, target atoms with unseen environments exhibited median errors approximately 3.0- to 3.5-fold larger than those with frequently represented environments. Taken together, these results provide a practical criterion for model use: predictions should be interpreted with caution when the target atom resides in a local atomic environment that is rare or absent in the training data, particularly when this is accompanied by low scaffold similarity.

### Conformational Impact on Prediction Accuracy for Uni-Mol-MeCAPs

We previously demonstrated that conformationally sensitive QM-derived properties were accurately predicted by the Uni-Mol models only when ground-truth (i.e., used for calculation of properties) conformers were used for model training and inference.^17^ Likewise, conformational effects on prediction accuracy were investigated for Uni-Mol-MeCAPs by testing three conformation types: xTB-optimized, force-field-optimized, and RMSD-max conformers. The xTB-optimized conformer was the one used to calculate true-MCA and MAA values (only for the substrate term), the force-field-optimized conformer was the one optimized under the MMFF94 force field, and the RMSD-max conformer was the most dissimilar to the xTB-optimized conformers in RMSD. To obtain the RMSD-max conformer for each compound, we first generated at most 200 diverse conformers using the ETKDGv3 generator with a pairwise RMSD of at least 2 Å, and then selected the conformer with the largest RMSD relative to the xTB-optimized one. Because the conformer generator in RDKit requires bond information, the *rdDetermineBonds* function was applied first. If the retrieved bonds did not match those of the input substrate, or no conformer had an RMSD of 2 Å or greater relative to the xTB-optimized structure, the compound was discarded. In this way, we obtained 21,633 conformers (compounds), and subsequent evaluations were conducted using this compound set.

The average RMSD between xTB-optimized and force-field-optimized was 2.31 Å with a standard deviation of 0.63 Å, and between xTB-optimized and the RMSD-max was 3.22 Å with a standard deviation of 0.55 Å. Compounds were divided into training, validation, and test sets according to the reference split, yielding data sets of 212,689 (training), 53,360 (validation), and 46,995 (test) for MCA and 173,350 (training), 43,367 (validation), and 38,428 (test) for MAA. Except for increasing the batch size to 128, all other training conditions were identical to those used for the other modeling approaches in this study.

The prediction accuracy of the Uni-Mol1-MeCAPs using the three conformation types as input is summarized in Table 4. The xTB-optimized conformation yielded the most accurate model, followed by the force-field-optimized and the RMSD-max conformations. The difference in RMSE between the xTB-optimized and the RMSD-max is 0.55 [kJ/mol] for MCA and 0.26 [kJ/mol] for MAA, suggesting that conformer quality consistently but slightly affects the prediction accuracy of the Uni-Mol1-MeCAPs.

**Table 4:** Accuracy Comparison for Inputting Different Conformers^a^

Target	Input conformer	Best epoch	*R* ^2^	Pearson *R*	RMSE [kJ/mol]
MCA	xTB-optimized	48	**0.98**	**0.99**	**10.58**
	Force field-optimized	50	**0.98**	**0.99**	10.99
	RMSD-max	50	**0.98**	**0.99**	11.13
MAA	xTB-optimized	50	**0.97**	**0.99**	**11.96**
	Force field-optimized	44	**0.97**	**0.99**	11.97
	RMSD-max	50	**0.97**	**0.99**	12.22

a The number of hidden layers in the regression head of Uni-Mol-MeCAPs was set to 0. The highest prediction accuracy values are in bold.

To examine whether local conformational variation is associated with changes in predictions, we quantified conformer-dependent error degradation and local geometric discrepancies around each reactive site. For each sample, we compared the reference xTB conformer with either the force field-optimized conformer (xTB-FF) or a deliberately perturbed conformer whose geometry was at least 2 Å away from the reference (xTB-RMSDmax). Conformer-dependent error degradation was defined as the change in absolute prediction error relative to the xTB-optimized reference conformer. For a given sample with experimental value *y*_*i*_, the degradation induced by an alternative conformer *C*∈{FF,RMSD max } was defined as

7 $$\Delta e_{i}^{\left(C\right)}=\left|{ŷ}_{i}^{\left(C\right)}-y_{i}\right|-\left|{\hat{y_{i}}}^{\left(\mathrm{xTB}\right)}-y_{i}\right|$$

where *ŷ*_*i*_^(*C*)^ and ${\hat{y_{i}}}^{\left(\mathrm{xTB}\right)}$ denote the predictions obtained from conformer *C* and the reference xTB conformer, respectively. Positive values of Δ*e*_*i*_^(*C*)^ therefore indicate deterioration in prediction accuracy relative to the xTB reference. In parallel, we quantified the local structural difference around the reaction site by the local pairwise-distance change, *D*_*i*_. For the local atom set *N*_*i*_, defined as the atoms within two bonds of the target site, *D*_*i*_ was calculated as

8 $$D_{i}^{\left(C\right)}=\sqrt{\frac{1}{\left|P_{i}\right|}\sum_{\left(u,v\right)\in P_{i}}{\left(d_{\mathrm{uv}}^{\left(\mathrm{xTB}\right)}-d_{\mathrm{uv}}^{\left(C\right)}\right)}^{2}}$$

where *P*_*i*_ is the set of all atom pairs in *N*_*i*_, and *d*_*uv*_^(*X*)^ is the interatomic distance between atoms *u* and *v* in conformers *X*. Because conformer-induced error changes were concentrated near zero for most samples, whereas substantial positive degradation was observed only in a subset of cases, median or average error changes remained small and did not clearly capture conformational effects (heatmap of median error changes is shown in Figure S2). We therefore focused on the 90th percentile Δ*e*, which more directly reflects the upper-tail deterioration induced by local conformational distortion.

We stratified the analysis by functional group and restricted the visualization to the five most frequent classes in each task (Figure 5). This chemically stratified view revealed that conformational sensitivity was not uniform across chemical environments. In MCA, amine-containing sites showed a clear increase in the 90th percentile with increasing *D* in both xTB-FF and xTB-RMSDmax, and this trend persisted even in higher-*D* bins that retained substantial sample counts, suggesting that amine-like environments are comparatively conformation-sensitive. A similar, although somewhat broader, tendency was observed for sites classified as atoms with lone pairs (atoms_with_lone_pair in Figure 5). For pyridine-like nitrogen atoms, error differences were high over all *D* bins for xTB-RMSDmax, suggesting that global conformational differences were a cause of deterioration. This could be explained by the involvement of large π-conjugated systems encompassing the atomic sites, which could not be captured by *D*. In MAA, sites classified as double bond (double_bond in Figure 5) and imine exhibited consistent increase in the 90th percentile across both conformer comparisons. Amide or ester-containing sites in MAA also showed a qualitatively similar pattern, except for bins with very limited sample counts for xTB-FF, and therefore appear likely to be conformation-sensitive as well. By contrast, some classes showed weaker or more moderate dependence on *D*, including ether-containing sites in MCA. Overall, xTB-RMSDmax showed an increasing trend in error differences as *D* increased. Globally and locally different conformations would deteriorate prediction accuracy.

**Figure 5:**
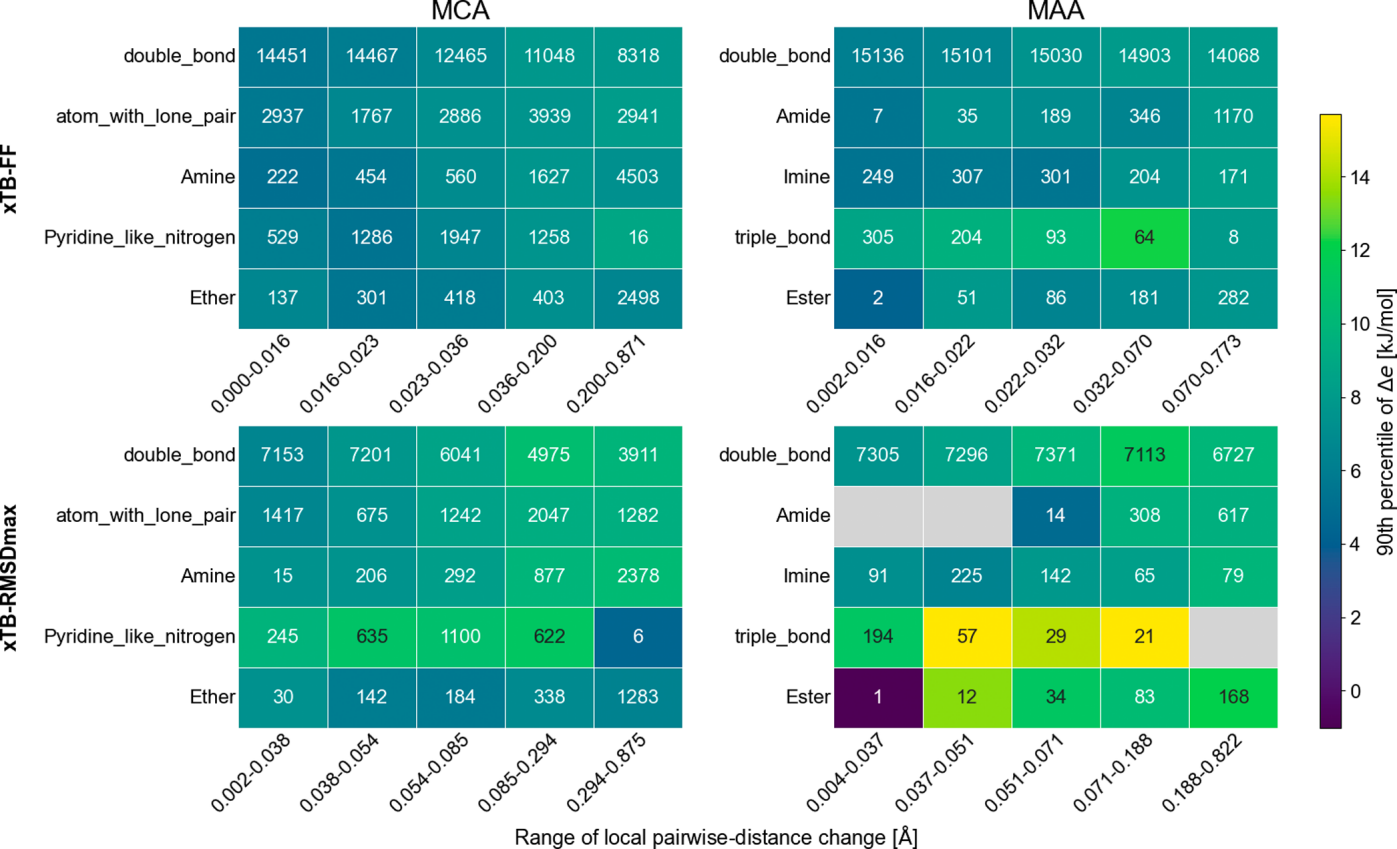
Functional-group-dependent conformational sensitivity. For each major functional-group class, samples were stratified by quantile bins of the local pairwise-distance change, D, and the 90th percentile of conformer-induced error degradation, Δ*e*, was calculated for the xTB-FF and xTB-RMSDmax comparisons. Numbers in the cells represent sample counts.

Since conformer-induced error changes were concentrated in a small range for most samples and the observation above was derived based on the 90th percentile of the error differences, we speculate that conformational dependence of MCA/MAA prediction is overall weak, but appreciable in specific functional groups or bonding environments when local geometric distortion is sufficiently large. These results indicate that force field-optimized conformers are generally adequate for routine MCA/MAA prediction, although additional caution is warranted in chemically sensitive environments such as amine-like sites in MCA and amide-like sites in MAA, where substantial local geometric distortion may lead to outliers. It should be noted that the conformational effects were generally smaller than those of modeling architecture selection, as shown in Table 2. However, using appropriate conformation improved prediction accuracy, particularly for the functional groups highlighted here.

### Protocol-to-Protocol Effects on MCA/MAA Regression

Using the prepared data set of 4827 compounds (training/validation/test = 3827/500/500) for QM protocol comparison, we trained the Uni-Mol1 and LightGBM models on the same fixed split and identical molecular sites. Unless noted otherwise, training conditions were identical to those used in the other modeling experiments in this study, except that we increased the maximum number of epochs to 300 for Uni-Mol1. The best Uni-Mol1 checkpoint was selected by validation performance. For LightGBM, we adopted the model formulation of Ree et al.^15^ on the same training/validation split. Performance was evaluated on the held-out test set (*n* = 500).

On the test set, Uni-Mol1 trained on the proposed r^2^SCAN-3c//GFN1-xTB reference achieved *R* ^2^ = 0.80 and MAE = 21.89 [kJ/mol] for MCA, and *R* ^2^ = 0.70 and MAE = 29.37 [kJ/mol] for MAA (top row of Figure 6). Across the QM reference protocols compared, performance differences were modest (Δ*R* ^2^ within 0.01; ΔMAE within 4 [kJ/mol]), indicating that—under this controlled same-site evaluation—the baseline electronic-structure protocol has a limited impact on the attainable regression accuracy when MMFF conformers are used as model inputs.

**Figure 6:**
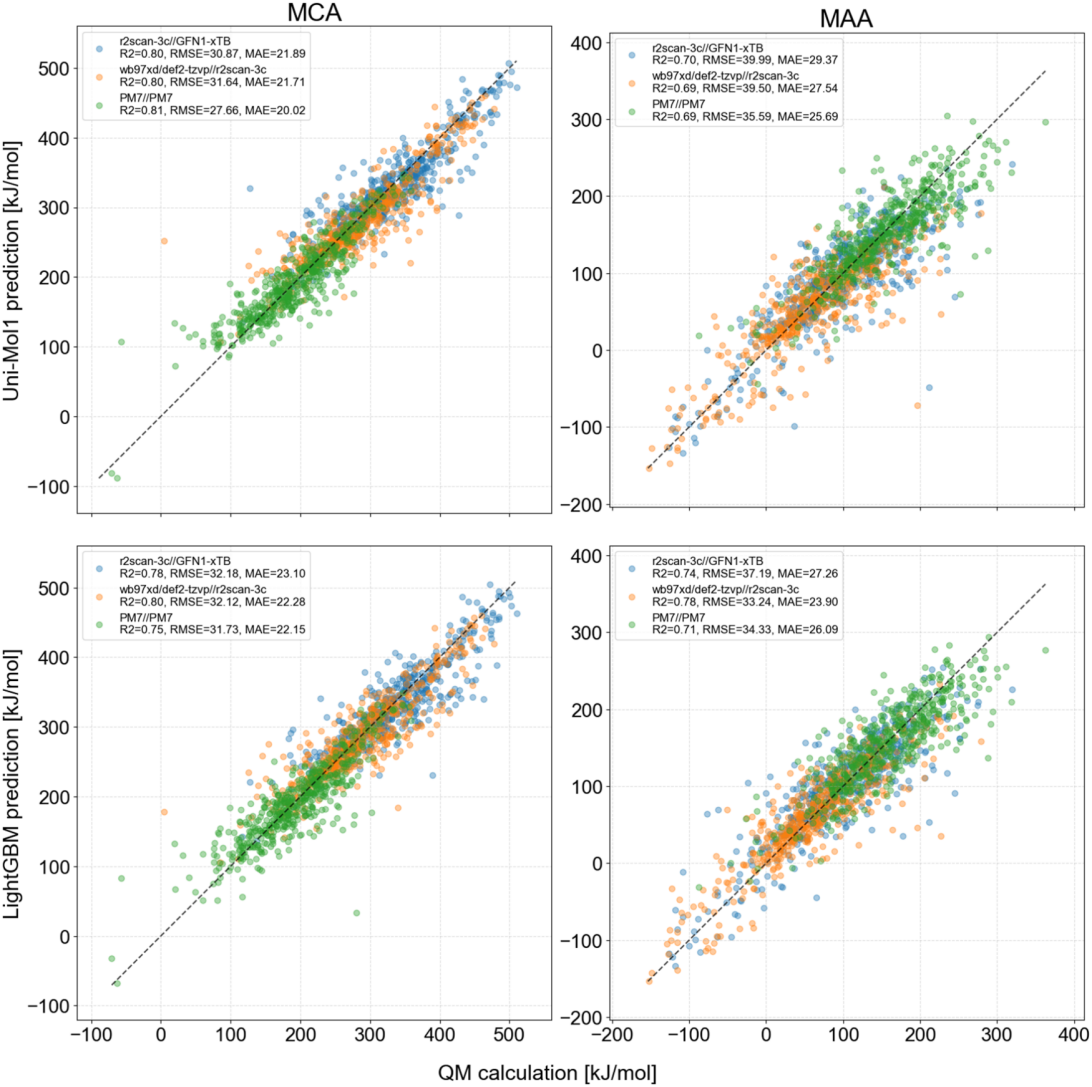
Protocol-to-protocol effects on MCA/MAA regression and comparison to descriptor-based LightGBM baselines. Predicted vs QM-calculated methyl cation affinity (MCA; left) and methyl anion affinity (MAA; right) on the held-out test set (*n* = 500), shown for Uni-Mol1 (top row) and LightGBM (bottom row).

Notably, the LightGBM models were competitive and, for MAA under protocols wB97-xd/def2-TZVP//r^2^SCAN-3c and r^2^SCAN-3c//GFN1-xTB, achieved lower errors than Uni-Mol1-MeCAP-An (bottom row of Figure 6). In this low-data regime, Uni-Mol1-MeCAPs, which used only MMFF conformer geometries and atom types as input without requiring any auxiliary QM calculations or handcrafted charge-feature engineering, may not fully optimize its parameters, unlike LightGBM with the CM5 charge model.

### Fine-Tuning Uni-Mol-MeCAPs to Higher QM Protocol Labeling

To further demonstrate protocol transferability, we conducted a transfer-learning experiment in which the target QM definition of MCA/MAA was changed while the train/validation/test split was kept fixed (3,827/500/500). We fine-tuned the Uni-Mol1-MeCAPs toward the higher-level DFT reference scheme (ωB97X-[D]/def2-TZVP//r^2^SCAN-3c, SMD­(DMSO)) by loading the weights of the Uni-Mol1 model (no hidden layer) previously trained on the full data set reported by Ree et al.,^15^ and then optimizing the all model parameters on the training portion of our protocol-benchmark data set by selecting the best epoch based on the validation set. All settings were kept consistent with the protocol-sensitivity benchmark. The resulting fine-tuned model achieved near-saturation performance on the held-out DFT-labeled test set (*R* ^2^ = 0.98 and MAE = 6.19 [kJ/mol] for MCA; *R* ^2^ = 0.98 and MAE = 7.06 [kJ/mol] for MAA, Figure 7), supporting that the proposed pipeline can be readily adapted to alternative (higher-level) electronic-structure reference schemes with a relatively small amount of protocol-specific labels.

**Figure 7:**
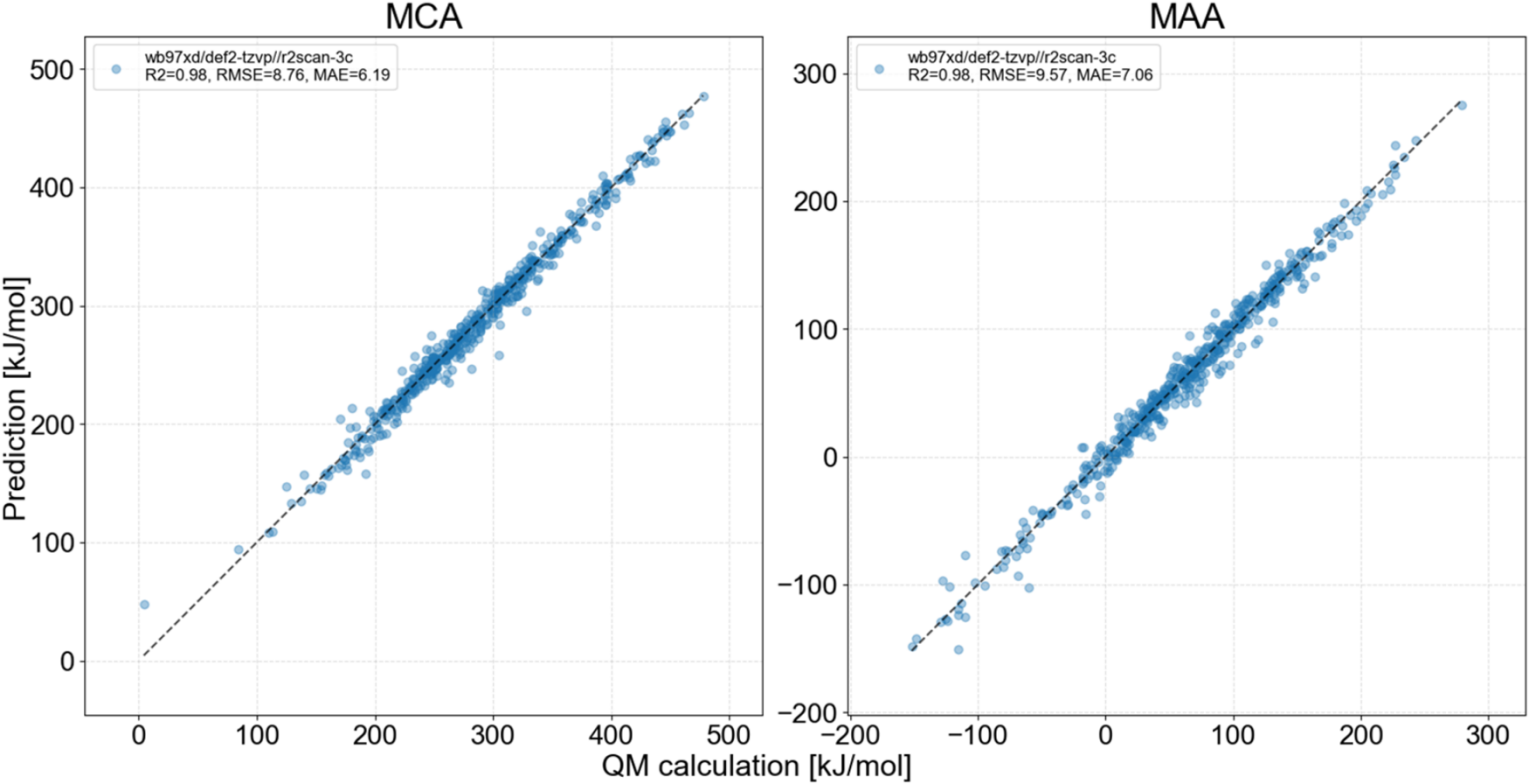
Protocol transferability through fine-tuning to a higher-level DFT reference. Predicted vs QM-calculated MCA (left) and MAA (right) on the held-out test set (*n* = 500) for the model fine-tuned to the higher-level DFT protocol (ωB97X-[D]/def2-TZVP//r^2^SCAN-3c, SMD­(DMSO)).

### Hidden Layer Optimization for the FFN in Uni-Mol-MeCAPs

We also aimed to optimize the number of hidden layers in the FFN, which outputs scaled MCA and MAA values. The prediction accuracies on the test data set using the reference split are summarized in Table 5 with the number of hidden layers ranging from 0 to 2. Apparently, there were no significant differences in prediction accuracy across layer depths in the FFN. A shallow network without hidden layers achieved high accuracy, possibly because the Uni-Mol encoder blocks were the primary contributors to this performance.

**Table 5:** Prediction Accuracy for the Test Dataset of the Reference Split Using Different Layer Depths (from Zero to Two) in the FFN part of the Uni-Mol-MeCAPs

Target	Model	Number of hidden layers	Best epoch	*R* ^2^	Pearson R	RMSE [kJ/mol]
MCA	Uni-Mol1-MeCAP-Cat	0	48	0.98	0.99	9.87
		1	50	0.98	0.99	**9.81**
		2	47	0.98	0.99	9.83
	Uni-Mol2-MeCAP-Cat	0	50	0.99	0.99	8.90
		1	36	0.99	0.99	**8.80**
		2	43	0.98	0.99	8.97
MAA	Uni-Mol1-MeCAP-An	0	48	0.98	0.99	10.99
		1	50	0.98	0.99	**10.95**
		2	37	0.98	0.99	11.10
	Uni-Mol2-MeCAP-An	0	50	0.98	0.99	**10.02**
		1	48	0.98	0.99	10.04
		2	50	0.98	0.99	10.06

### Demonstration: Reactive Site Prioritization by Predicted MCA/MAA Ranking

To examine the applicability of the Uni-Mol-MeCAPs for practical reaction-center analysis, we conducted a demonstration using reaction data from ChemDB,^39^ which comprises 100 polar reactions with mechanisms, including reaction SMIRKS and local electron-flow information. Following the reaction annotations, we focused on atomic sites involved in the formation of new σ-bonds and excluded cases involving single pericyclic-like π-systems. For each reaction, the nucleophilic source atom and electrophilic sink atom were identified based on the annotated electron flow, and the source MCA and sink MAA were used as site-reactivity descriptors. Reactions were removed in which either the source or sink could not be recognized as an MCA/MAA site, and quantum-chemical evaluation failed. Because the training data sets used for both Uni-Mol-MeCAPs and LightGBM with CM5 charge models consisted exclusively of neutral molecules, we extracted reactions whose reactant-side species contained no formally charged atoms. Under these constraints, 13 reactions were retained for analysis. The number of eligible reactions in this analysis was limited, and the following results should be regarded as a demonstration of Uni-Mol-MeCAPs for reaction site prediction. We used Uni-Mol2-MeCAPs without an additional hidden layer. For QM-based reference reactivities, MCA and MAA values were derived using ESNUEL,^13^ the labeling QM protocol for MCA and MAA. Site rank correlations were evaluated only for eligible sites specified by ESNUEL that had no connectivity errors after methyl cation/anion attachment.

The distributions of reaction-wise Kendall’s τ correlation values between ESNUEL and the machine learning predictions are shown in Figure 8. For both MCA and MAA, both models showed generally high Kendall’s τ values, indicating that they were able to reproduce the overall within-reaction site-ordering trend reasonably. From the box positions and lower tails in Figure 8, Uni-Mol-MeCAPs appeared to show a slightly higher median and somewhat more stable behavior overall. Nevertheless, exact identification of the top-ranked ESNUEL site remained more favorable for the LightGBM models, with top-1 match rates of 1.00 for MCA and 0.92 for MAA, compared with 0.92 for MCA and 0.85 for MAA for Uni-Mol-MeCAPs.

**Figure 8:**
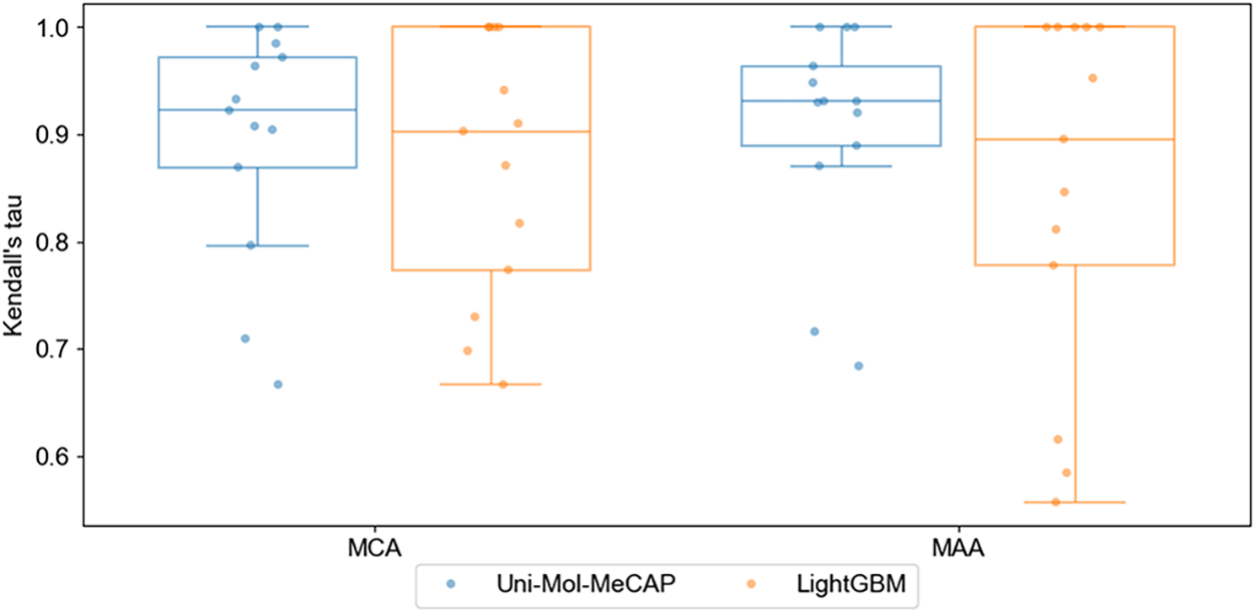
Distribution of reaction-wise Kendall’s τ values between ESNUEL-derived and model-predicted site rankings for MCA and MAA. For each reaction, the rank correlation was computed using only those candidate sites for which the ESNUEL calculations successfully returned valid values without connectivity errors.

Example reactions selected from cases with valid ESNUEL values are shown in Figure 9. In both MCA and MAA examples shown here, Uni-Mol-MeCAPs exhibited slightly higher reaction-wise Kendall’s τ values than the LightGBM with CM5 charge models baseline, while both models broadly reproduced the ESNUEL-derived ordering of the major competing sites, although for MAA, the LightGBM with CM5 charge model predicted MAA comparable values for atomic sites 1 and 9 in Reactant 0. For neutral-reactant systems, the differences between the two models may arise from subtle reordering among chemically similar candidate sites. In such cases, small score differences among competing sites may influence the final rank correlation, even when the major reactivity trend is qualitatively preserved.

**Figure 9:**
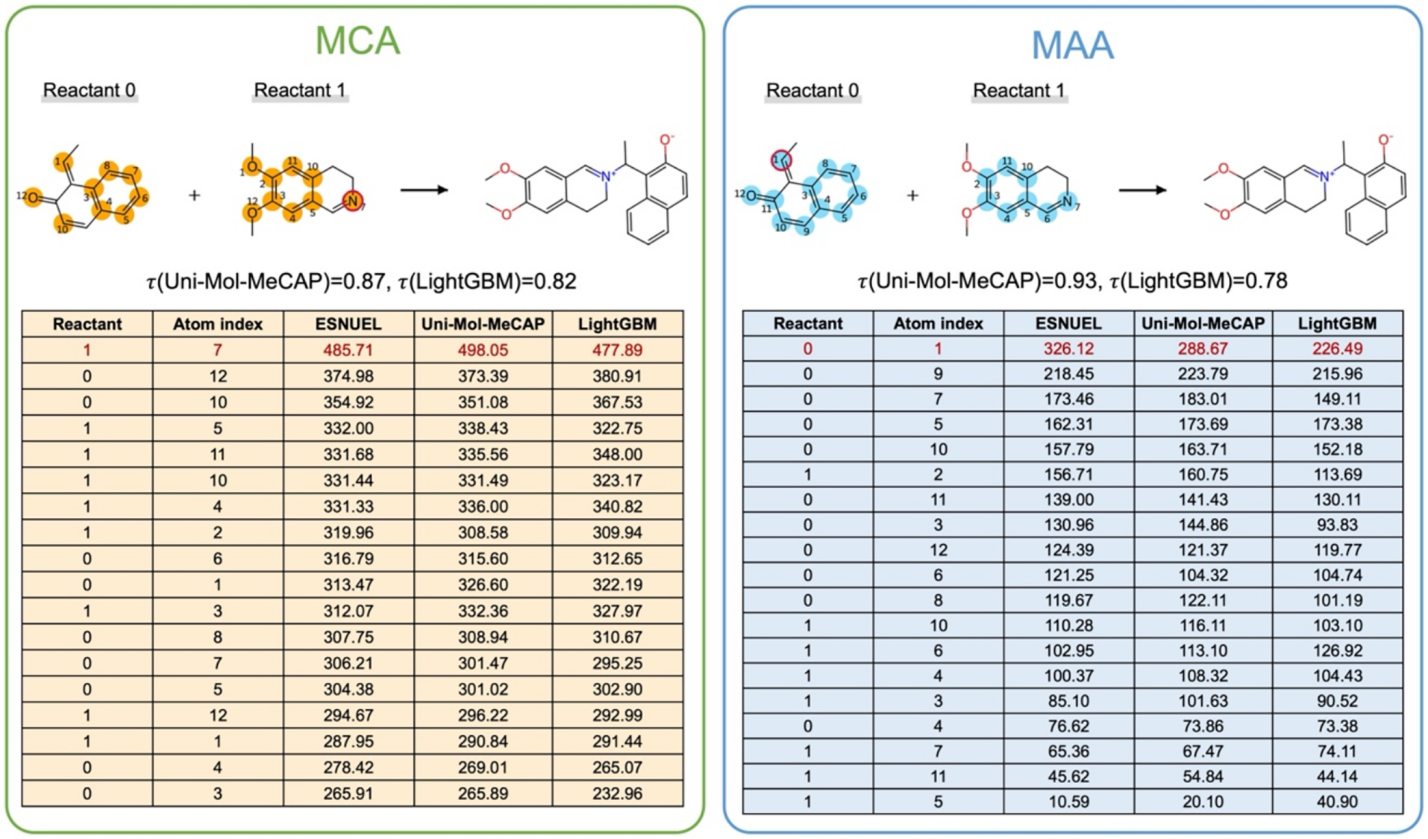
Reaction examples for MCA and MAA prediction. Red circles indicate the annotated reactive site (nucleophilic site for MCA and electrophilic site for MAA), and the numbers shown near the highlighted atoms denote atom indices. The tables list candidate sites in descending order of the ESNUEL-derived MCA/MAA values, along with the corresponding Uni-Mol-MeCAP and LightGBM predictions (Units: [kJ/mol]). The reaction-wise Kendall’s τ values shown below each reaction indicate the rank correlation between ESNUEL-derived and model-predicted within-reaction site orderings.

## Conclusions

Predicting site-specific nucleophilicity and electrophilicity is important for the design of polar reactions. Computationally calculable MCA and MAA are indicators of nucleophilicity and electrophilicity, respectively, and an efficient workflow for calculating them was previously proposed. Nevertheless, further reductions of computational cost were necessary to evaluate many molecules, and highly accurate surrogate models for MCA and MAA were required.

We introduce Uni-Mol-MeCAPs as surrogate models for MCA and MAA that utilize pretrained Uni-Mol encoder blocks. Comparing Uni-Mol architectures, Uni-Mol2 slightly but consistently outperformed Uni-Mol1 as an encoder block within the Uni-Mol-MeCAP models. The proposed Uni-Mol-MeCAP models achieved RMSEs of 8.90 [kJ/mol] for MCA and 10.02 [kJ/mol] for MAA, surpassing the previous state-of-the-art models (RMSE: 17.45 [kJ/mol] for MCA, RMSE: 22.04 [kJ/mol] for MAA). The importance of using three-dimensional information was indirectly supported by the low prediction accuracy of ChemProp models and comaprable prediction accuracy of Uni-Mol-MeCAPs without pretraining. Additionally, using xTB-optimized conformers as input to Uni-Mol1-MeCAPs yielded the highest prediction accuracy among the three conformer types: the xTB-optimized, force-field-optimized, and RMSD-max. Although differences in the accuracy of Uni-Mol1-MeCAPs among these conformers were marginal, using unstable conformers yielded outlier values for some compounds, underscoring the importance of employing a chemically meaningful conformation. Uni-Mol1-MeCAPs were also rigorous in the underlying QM protocols for MCA and MAA, although prediction accuracy decreased, likely due to a small number of training samples. However, this was overcome by fine-tuning the pretrained Uni-Mol-MeCAPs on a small number of yet more accurate MCA and MAA samples.

## Supplementary Material



## Data Availability

The codes for Uni-Mol-MeCAP training and for reproducing the figures and tables described in this work are freely available from the GitHub repository at https://github.com/iwmspy/MeCAP. The scripts for downloading and curating the data sets from references 15,39 are also available in the repository.

## Notes

Paired analysis to assess statistical significance (Section S1), relationship between training-set occurrence of target-centered hashes of Morgan fingerprint and prediction error (Figure S1), and heatmaps for functional-group-dependent conformational sensitivity using median Δ*e* (Figure S2) (PDF)
