# Supplementary material for: Methyl Cation Affinity and Methyl Anion Affinity Prediction Using Uni-Mol-Based Models
Source: J Phys Chem A. 2026 Jun 16;130(26):5016–29. doi: 10.1021/acs.jpca.5c08537 (PMC13339650; doi:10.1021/acs.jpca.5c08537)
Supplement: Supplementary file 1 [file jp5c08537_si_001.pdf]

Supporting Information

for

Methyl Cation Affinity and Methyl Anion Affinity Prediction Using Uni-Mol-based Models

Yuto Iwasaki,[1] Akinori Sato[2,1], Tomoyuki Miyao[2,1]*

[1]Graduate School of Science and Technology, Nara Institute of Science and Technology, 8916-5 Takayama-cho, Ikoma, Nara, 630-0192, Japan.

[2]Data Science Center, Nara Institute of Science and Technology, 8916-5 Takayama-cho, Ikoma, Nara, 630-0192, Japan.

*Corresponding author:

E-mail: miyao@dsc.naist.jp

Section S1. Paired analysis to assess statistical significance

To compare the prediction errors of the Uni-Mol1- and Uni-Mol2-based models on the same held-out test set, we performed a paired analysis using the per-sample absolute errors. For each test sample i, the absolute errors of the two models were calculated as $|y_i - \hat{y}_{i,\mathrm{Uni-Mol1}}|$ and $|y_i - \hat{y}_{i,\mathrm{Uni-Mol2}}|$. The per-sample paired difference was then defined by **eq. S1**:

$$d_i = |y_i - \hat{y}_{i,\mathrm{Uni-Mol1}}| - |y\_i - \hat{y}_{i,\mathrm{Uni-Mol2}}| \qquad \text{(S1)},$$

so that a positive value indicates a smaller absolute error for the Uni-Mol2-based model.

A paired t-test was then applied to these per-compound differences to test whether their mean was different from zero. The 95% confidence interval for the mean paired difference was calculated using the t distribution with $N - 1$ degrees of freedom, where N is the number of test compounds.

Supplemental figures

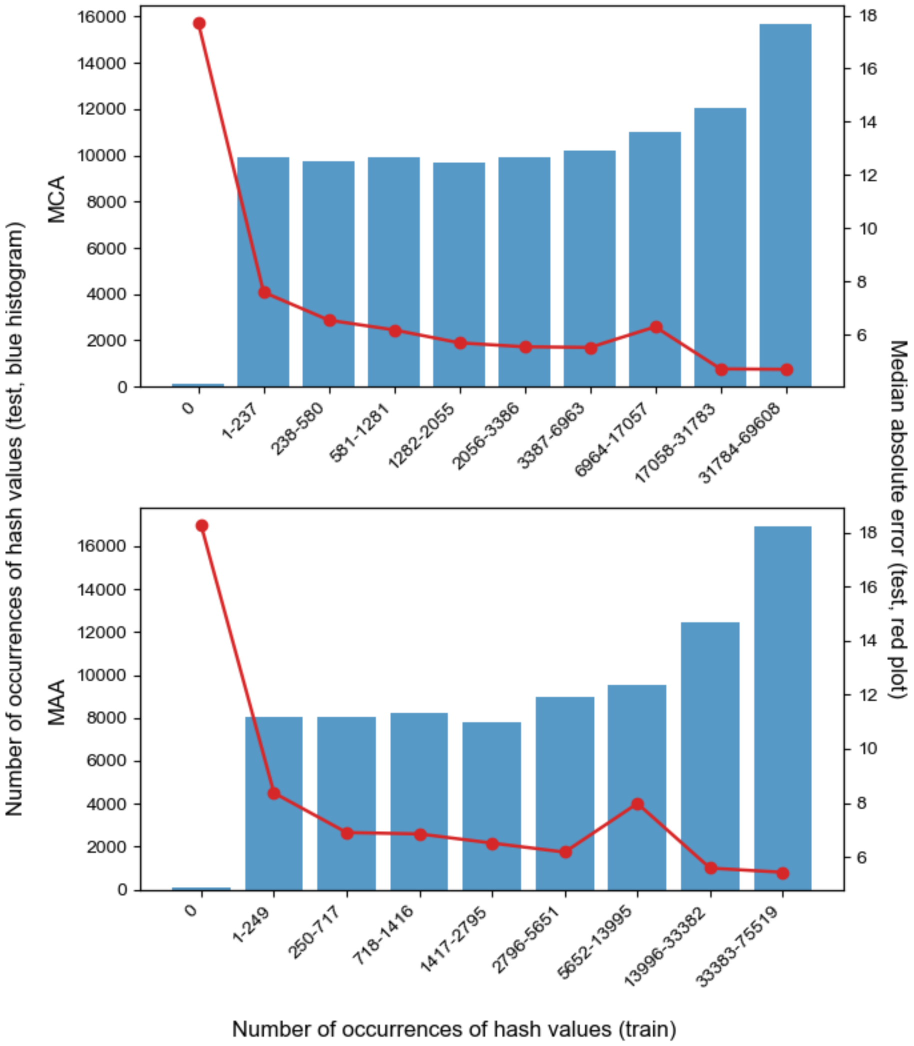

Figure S1 Relationship between training-set occurrence of target-centered hashes of Morgan fingerprint and prediction error. For each test sample, a radius-1 Morgan environment hash was generated from the target atom, and samples were grouped according to the number of occurrences of the same hash in the training set. Blue bars indicate the number of test samples in each occurrence bin, and red points indicate the median absolute error for the corresponding bin. Results are shown separately for MCA and MAA.

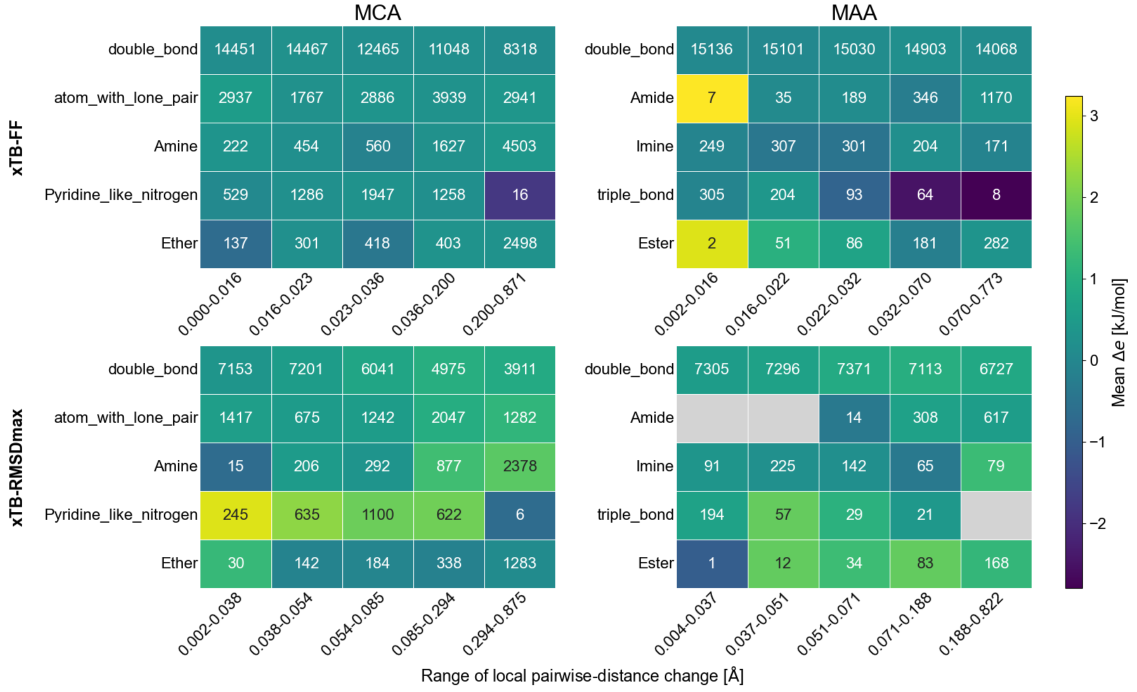

Figure S2. Heat maps for functional-group-dependent conformational sensitivity using median Δe. For each major functional-group class, samples were stratified by quantile bins of the local pairwise-distance change, D, and the average of conformer-induced error degradation, Δe, was calculated for the xTB-FF and xTB-RMSDmax comparisons. Numbers in the cells represent sample counts.
